# Concurrent validity, cut‐offs and ability to change of patient‐reported outcome measures for rhinitis and asthma in MASK‐air^®^

**DOI:** 10.1002/clt2.12390

**Published:** 2024-09-23

**Authors:** Jean Bousquet, Bernardo Sousa‐Pinto, Josep M. Anto, Anna Bedbrook, Wienczyslawa Czarlewski, Ignacio J. Ansotegui, Karl‐C. Bergmann, Fulvio Braido, Luisa Brussino, Lorenzo Cecchi, Claudia Chaves Loureiro, Alvaro A. Cruz, Philippe Devillier, Alessandro Fiocchi, Bilun Gemicioglu, Tari Haahtela, Juan Carlos Ivancevich, Ludger Klimek, Marek Kulus, Piotr Kuna, Maciej Kupczyk, Violeta Kvedariene, Desiree E. Larenas‐Linnemann, Gilles Louis, Renaud Louis, Michael Makris, Mario Morais‐Almeida, Marek Niedoszytko, Ken Ohta, Markus Ollert, Nikolaos Papadopoulos, Vincenzo Patella, Benoit Pétré, Oliver Pfaar, Francesca Puggioni, Santiago Quirce, Frederico S. Regateiro, Nicolas Roche, Philip W. Rouadi, Boleslaw Samolinski, Joaquin Sastre, Florence Schleich, Nicola Scichilone, Luis Taborda‐Barata, Sanna Toppila‐Salmi, Arunas Valiulis, Ilgim Vardaloglu Koyuncu, Maria Teresa Ventura, Arzu Yorgancioglu, Joao A. Fonseca, Torsten Zuberbier

**Affiliations:** ^1^ Institute of Allergology Charité – Universitätsmedizin Berlin Corporate Member of Freie Universität Berlin and Humboldt‐Universität zu Berlin Berlin Germany; ^2^ Fraunhofer Institute for Translational Medicine and Pharmacology ITMP Immunology and Allergology Berlin Germany; ^3^ ARIA Montpellier France; ^4^ MEDCIDS ‐ Department of Community Medicine Information and Health Decision Sciences Faculty of Medicine University of Porto Porto Portugal; ^5^ CINTESIS@RISE– Health Research Network Faculty of Medicine University of Porto Porto Portugal; ^6^ ISGlobal Barcelona Institute for Global Health Barcelona Spain; ^7^ Universitat Pompeu Fabra (UPF) Barcelona Spain; ^8^ CIBER Epidemiología y Salud Pública (CIBERESP) Barcelona Spain; ^9^ Medical Consulting Czarlewski Levallois France; ^10^ Department of Allergy and Immunology Hospital Quironsalud Bizkaia Bilbao Spain; ^11^ Respiratory and Allergy Clinic, IRCCS ‐ Policlinico San Martino and Department of Internal Medicine (DIMI), University of Genoa Genoa Italy; ^12^ Department of Medical Sciences University of Torino Torino Italy; ^13^ Allergy and Clinical Immunology Unit Mauriziano Hospital Torino Italy; ^14^ SOS Allergology and Clinical Immunology USL Toscana Centro Prato Italy; ^15^ Department of Pneumology and University of Coimbra Medicine Faculty Coimbra Portugal; ^16^ Coimbra Institute for Clinical and Biomedical Research CIBB Coimbra Portugal; ^17^ Fundaçao ProAR Federal University of Bahia and GARD/WHO Planning Group Salvador Bahia Brazil; ^18^ VIM Suresnes UMR 0892 Pôle des Maladies des Voies Respiratoires Hôpital Foch Université Paris‐Saclay Suresnes France; ^19^ Allergy Bambino Gesù Children's Hospital Istituto di Ricovero e Cura a Carattere Scientifico (IRCCS) Rome Italy; ^20^ Department of Pulmonary Diseases and Institute of Pulmonology and Tuberculosis Istanbul University‐Cerrahpaşa Cerrahpaşa Faculty of Medicine Istanbul Turkey; ^21^ Skin and Allergy Hospital, Helsinki University Hospital, and University of Helsinki Helsinki Finland; ^22^ Servicio de Alergia e Immunologia Clinica Santa Isabel Buenos Aires Argentina; ^23^ Department of Otolaryngology Head and Neck Surgery Universitätsmedizin Mainz Mainz Germany; ^24^ Center for Rhinology and Allergology Wiesbaden Germany; ^25^ Department of Pediatric Respiratory Diseases and Allergology Medical University of Warsaw Warsaw Poland; ^26^ Division of Internal Medicine Asthma and Allergy Barlicki University Hospital Medical University of Lodz Lodz Poland; ^27^ Institute of Clinical Medicine Clinic of Chest Diseases and Allergology Faculty of Medicine Vilnius University Vilnius Lithuania; ^28^ Department of Pathology Institute of Biomedical Sciences Faculty of Medicine Vilnius University Vilnius Lithuania; ^29^ Center of Excellence in Asthma and Allergy Médica Sur Clinical Foundation and Hospital México City Mexico; ^30^ Department of Public Health University of Liège Liège Belgium; ^31^ Department of Pulmonary Medicine CHU Liège Liège Belgium; ^32^ GIGA I3 Research Group University of Liège Liège Belgium; ^33^ Allergy Unit “D Kalogeromitros” 2nd Department of Dermatology and Venereology National & Kapodistrian University of Athens “Attikon” University Hospital Athens Greece; ^34^ Allergy Center CUF Descobertas Hospital Lisbon Portugal; ^35^ Department of Allergology Medical University of Gdańsk Gdansk Poland; ^36^ Japan Antituberculosis Association (JATA) Fukujuji Hospital Tokyo Japan; ^37^ National Hospital Organization Tokyo National Hospital, and JATA Fukujuji Hospital Tokyo Japan; ^38^ Department of Infection and Immunity Luxembourg Institute of Health Esch‐sur‐Alzette Luxembourg; ^39^ Department of Dermatology and Allergy Center Odense Research Center for Anaphylaxis (ORCA) Odense University Hospital Odense Denmark; ^40^ Allergy Department 2nd Pediatric Clinic University of Athens Athens Greece; ^41^ Division of Allergy and Clinical Immunology Department of Medicine “Santa Maria della Speranza” Hospital Salerno Italy; ^42^ Agency of Health ASL Salerno Italy; ^43^ Postgraduate Programme in Allergy and Clinical Immunology University of Naples Federico II Naples Italy; ^44^ Section of Rhinology and Allergy Department of Otorhinolaryngology Head and Neck Surgery University Hospital Marburg Philipps‐Universität Marburg Marburg Germany; ^45^ IRCCS Humanitas Research Center Personalized Medicine Asthma & Allergy Milan Italy; ^46^ Department of Allergy Hospital La Paz Institute for Health Research (IdiPAZ) Madrid Spain; ^47^ Allergy and Clinical Immunology Department, Hospitais da Universidade de Coimbra, Unidade Local de Saúde de Coimbra Coimbra Portugal; ^48^ Center for Innovative Biomedicine and Biotechnology (CIBB), Faculty of Medicine University of Coimbra Coimbra Portugal; ^49^ Institute of Immunology Faculty of Medicine University of Coimbra Coimbra Portugal; ^50^ Pneumologie AP‐HP Centre Université de Paris Cité Hôpital Cochin Paris France; ^51^ UMR 1016 Institut Cochin Paris France; ^52^ Department of Otolaryngology‐Head and Neck Surgery Eye and Ear University Hospital Beirut Lebanon; ^53^ Department of Otorhinolaryngology‐Head and Neck Surgery Dar Al Shifa Hospital Salmiya Kuwait; ^54^ Department of Prevention of Environmental Hazards Allergology and Immunology Medical University of Warsaw Warsaw Poland; ^55^ Allergy Service Fundacion Jimenez Diaz Autonoma University of Madrid CIBERES‐ISCIII Madrid Spain; ^56^ PROMISE Department University of Palermo Palermo Italy; ^57^ Department of Immunoallergology Cova da Beira University Hospital Centre Covilhã Portugal; ^58^ UBIAir ‐ Clinical & Experimental Lung Centre and CICS‐UBI Health Sciences Research Centre University of Beira Interior Covilhã Portugal; ^59^ Department of Otorhinolaryngology University of Eastern Finland and the North Savo Wellbeing Services County Kuopio Finland; ^60^ Department of Allergy Inflammation Center Skin and Allergy Hospital, Inflammation Center, Helsinki University Hospital and University of Helsinki Helsinki Finland; ^61^ Clinic of Asthma, Allergy, and Chronic Lung Diseases Vilnius Lithuania; ^62^ Interdisciplinary Research Group of Human Ecology, Institute of Clinical Medicine, Institute of Health Sciences, and Clinic of Children's Diseases, Medical Faculty of Vilnius University Vilnius Lithuania; ^63^ University of Bari Medical School Bari Italy; ^64^ Institute of Sciences of Food Production National Research Council (ISPA‐CNR) Bari Italy; ^65^ Department of Pulmonary Diseases Celal Bayar University Faculty of Medicine Manisa Turkey

**Keywords:** asthma, digital health, EQ‐5D, rhinitis, visual analogue scale

## Abstract

Patient‐reported outcome measures (PROMs) are used to assess a patient's health status at a particular point in time. They are essential in the development of person‐centred care. This paper reviews studies performed on PROMs for assessing AR and asthma control, in particular VAS scales that are included in the app MASK‐air^®^ (Mobile Airways Sentinel networK) for asthma and rhinitis. VASs were initially developed on paper and pencil and tested for their criterion validity, cut‐offs and responsiveness. Then, a multicentric, multinational, double‐blind, placebo‐controlled, randomised control trial (DB‐PC‐RCT) using an electronic VAS form was carried out. Finally, with the development of MASK‐air^®^ in 2015, previously validated VAS questions were adapted to the digital format and further methodologic evaluations were performed. VAS for asthma, rhinitis, conjunctivitis, work and EQ‐5D are included in the app. Additionally, two control‐medication scores for allergic symptoms of asthma (e‐DASTHMA) were validated for their criterion validity, cut‐offs and responsiveness.

## 1 INTRODUCTION

Patient‐reported outcomes (PROs) are increasingly used in routine practice to enhance clinical care by improving shared‐decision making, clinician awareness of symptoms, symptom management, patient satisfaction and quality of life.^1^ PROs must be carefully defined to capture important information from patients. This information should be measured accurately to make it comparable with other measurements. PROs may concern signs and symptoms, physical functioning domains (e.g., sleep), social functioning domains (e.g., limitations in school and work), among others.^2^ In rhinitis or asthma, several PROMs (PRO measures) are used including visual analogue scales (VASs), total symptom scores (Box 1), combined symptom‐medication scores, quality‐of‐life or objective measurements (e.g. pulmonary function tests).

This paper reviews studies performed on PROMs for assessing AR and asthma control, in particular VAS scales. We will start by discussing the overall advantages and drawbacks of VAS for AR and asthma, followed by a review of initial studies carried out on paper and pencil, then using electronic diaries and finally using the MASK‐air^®^ app. The same questions (translated into more than 20 languages) were used in all studies.

## 2 OVERALL ASSESSMENT OF VAS IN RHINITIS AND ASTHMA

### 2.1 Overall assessment of VAS

The VAS is a psychometric response scale used in questionnaires for measuring subjective characteristics or attitudes that cannot be directly measured. It has strengths and weaknesses (Table 1).

**TABLE 1:** Strengths and weaknesses of the VAS.

Strengths The VAS is more sensitive to small changes than simple descriptive ordinal scales on which symptoms are rated, for example, as mild, moderate or severe. The VAS takes less than 1 minute to complete. It is easy to use with routine treatment. Training is not usually required to fill in a VAS, but some training may be needed when it is administered digitally. It is acceptable for the patients. It is inexpensive. Weaknesses The assessment is clearly highly subjective. The VAS may be less valuable when comparing a group of patients at a one‐time point. The VAS is administered either as a paper‐and‐pencil measure or digitally. As a result, it cannot be administered verbally or by phone.^3^ Test–retest reliability is good, but is higher among literate compared to illiterate patients^4^ and among younger compared to older adults. Drawbacks with paper‐filled VASs do not exist when administered digitally. In some diseases, in the absence of a gold standard, criterion validity cannot be evaluated.

### 2.2 VAS in asthma and rhinitis in MASK‐air^®^: From paper and pencil to mHealth

The questions included in any VAS should be developed and validated to represent a PROM easily understood by the patient in many different languages and cultures. On the other hand, the number of questions should be limited in a tool.

The first studies with VASs in AR and asthma were based on the paper‐and‐pencil format. Regarding pain, a single and very simple question was largely used to assess the severity and control of pain under treatment.^5^, ^6^, ^7^, ^8^ In MASK‐air^®^, the same simple approach was used. We did not use individual symptoms of rhinitis and asthma because their pooling to propose a score should be validated using complex formulae to ascribe to each of the symptoms (e.g., rhinorrhoea, sneezing, nasal obstruction and/or pruritus) a weight in the burden and/or perception of the disease that may differ with age.^9^, ^10^

VASs were then developed using paper and pencil and tested for their criterion validity, cut‐offs and responsiveness. Then, a multicentric, multinational DB‐PC‐RCT using an electronic VAS form was carried out (Table 2). Finally, with the development of MASK‐air^®^ (Mobile Airways Sentinel networK) in 2015,^24^ previously validated VAS questions (Table 3) were adapted to the digital format and further methodologic evaluations were performed.

**TABLE 2:** Studies performed to validate the MASK‐air^®^ VASs.

Study		Country	*N* patients	VAS	Type of study	Comparator		
						RQLQ	TNSS/TSS	Other
1	Paper^11^	France	3052	Global, asthma^a^	Observational	X		ARIA
2	Paper^12^, ^13^, ^14^	France	586	Global, nose, asthma^a^	Cluster RCT	X	X	
3	Paper^15^, ^16^, ^17^	France	990	Global	Observational	X	X	ARIA
4	Paper^9^, ^10^	France	806	Global	Observational	X	X	Work
5	Paper^18^, ^19^, ^20^	Japan	29,518	Nose	Observational			GINA
				Asthma				ACT
6	e‐diary^21^	Europe‐CDN	547	Global	DB‐PC‐RCT	X	X	
7	e‐diary^22^	Europe‐CDN	716	Global	DB‐PC‐RCT	X	X	
8	e‐diary^23^	Europe	482	Rhino‐conjunctivitis	DB‐PC‐RCT	X	X	

Abbreviations: ACT, asthma control test; ARIA, Allergic Rhinitis and its Impact on Asthma; CDN, Canada; DB‐PC, double‐blind, placebo‐controlled; RCT, randomised control trial.

^a^ Unpublished.

**TABLE 3:** Visual analogue scales used for the daily monitoring of symptoms and impact in all studies and MASK‐air^®^.

Scale	Question
Daily monitoring	
VAS global allergy symptoms	‘Overall, how much are your allergic symptoms bothering you today?’
VAS nose	‘How much are your nose symptoms bothering you today?’
VAS eyes	‘How much are your eye symptoms bothering you today?’
VAS asthma	‘How much are your asthma symptoms bothering you today?’
Impact	
VAS work	‘How much are your allergic symptoms affecting your work today?’
VAS education^a^	‘Today, how much did allergies affect your productivity while in school or attending classes in an academic setting?’
EQ‐5D‐5L VAS	EQ‐5D‐5L VAS^25^

^a^ Question derived from WPAI‐AS^10^, ^26^, ^27^.

We also added to MASK‐air^®^ the impact of the diseases on work^28^ or education^29^ as well as a generic VAS QOL questionnaire (EQ‐5D‐5L VAS).^25^, ^30^ One question, “dyspnoea,” was added temporarily to MASK‐air^®^ but the results showed a highly significant correlation with VAS ‘asthma’ in patients with severe asthma (rho = 0.93 and 0.90).^31^, ^32^ The question was therefore subsequently deleted.

We used either the total symptom score (TNSS, TOS or TSS), a validated QOL measure (RQLQ) or an asthma control score as gold standards to assess VAS construct validity in pre‐MASK‐air^®^ studies (Table 2). In MASK‐air^®^, we used the impact on work or academic productivity or utilities (EQ‐5D‐5L) as gold standards. In addition, we studied cut‐off values and the ability to change before and during MASK‐air^®^ studies.

## 3 INITIAL STUDIES USING VAS ‘GLOBAL ALLERGY SYMPTOMS’ ON PAPER AND PENCIL IN FRANCE

### 3.1 VAS in ARIA classes in an observational study in primary care (2004)

In study 1, Allergic Rhinitis and its Impact on Asthma (ARIA)^33^ classes could be differentiated by VAS in AR patients seen in primary care.^11^ VAS scores ranged from not at all bothersome (0 cm) to extremely bothersome (10 cm). ARIA severity had more impact on VAS levels than duration. The receiver operating characteristic (ROC) curve identified a 5 cm cut‐off for patients classified as 'mild' or ‘moderate/severe’ rhinitis (negative predictive value: 93.5; positive predictive value: 73.6%). RQLQ global scores were moderately but significantly correlated (rho = 0.46; *p* < 0.0001).

### 3.2 VAS in a multi‐centre, cluster‐randomised trial in specialists (2005)

**FIGURE 1:**
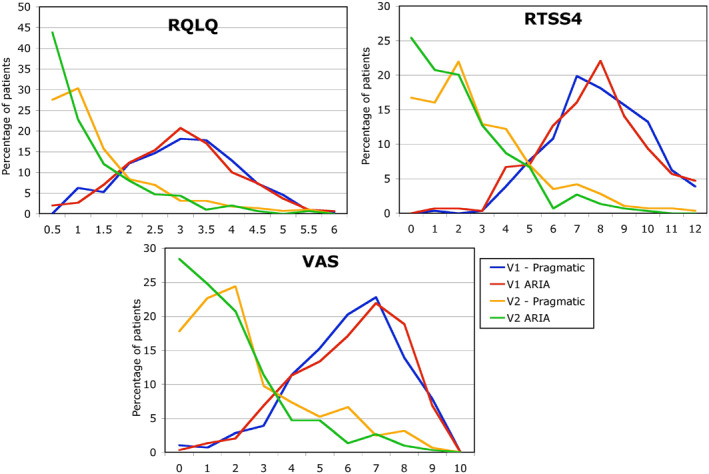
VAS, RQLQ and TNSS before and after treatment (from^14^).

In study 2, in patients with grass pollen‐induced AR seen in specialist care in France,^12^ a multicentre, cluster‐randomised trial compared two therapeutic strategies. The RQLQ, the TNSS and VAS (0–10 cm) were self‐reported by the patients before and after two weeks of treatment. A first *post hoc* analysis using ROC curves found that the optimal cut‐off in VAS change separating patients without improvement from those with improvement was 0.30 cm.^13^ By using cost function, a difference of more than 1 cm was significant. A second *post hoc* analysis compared TNSS, VAS and RQLQ before and after treatment.^12^, ^14^ (Figure 1 and Table 4). VAS, RQLQ and TNSS showed similar change patterns and confirmed the ability to change VAS.

**TABLE 4:** Efficacy of the two treatment strategies (from^14^).

		Free‐choice treatment (pragmatic)	VAS‐based strategy (ARIA)
VAS	V1	6.5 (5.0–7.7)	6.8 (5.0–8.0)
	V2	2.0 (1.0–4.0)^a^	1.7 (0.7–3.0)^b^
RQLQ global score	V1	2.8 (2.0–3.5)	2.7 (2.0–3.4)
	V2	0.9 (0.4–1.6)^a^	0.6 (0.3–1.3)^b^
TNSS	V1	8.0 (7.0–9.0)	8.0 (6.0–9.0)
	V2	2.0 (1.0–4.0)^a^	2.0 (0.0–3.0)^a^

*Note*: V1: visit at baseline, V2: visit after 2 weeks of treatment. Results in medians and 25%–75 %.

Abbreviations: ARIA, Allergic Rhinitis and its Impact on Asthma; RQLQ, Rhinoconjunctivitis Quality of Life Questionnaire; TNSS, Total Nasal Symptom Score; VAS, Visual analogue scale.

^a^ *p* < 0.001 by comparison to V1.

^b^ *p* < 0.001 by comparison to V1 and *p* < 0.01 by comparison to Free‐treatment choice.

### 3.3 VAS levels in a multi‐centre observational study in primary care (2011)

The third study performed on patients consulting in primary care for AR assessed the impact of AR symptoms and the ARIA classes on QoL.^15^ On day 14, treatments did not affect VAS levels. Changes in VAS levels were similar to those in TNSS4 and slightly lower than those in TSS6 or RQLQ. **An** a priori **sub‐analysis** validated VAS as a simple quantitative tool to assess the burden of AR^16^ and identified cut‐off VAS levels for discriminating clinically relevant changes. The VAS cut‐off variation of 23/100 mm was associated with a variation of 0.5 for RQLQ (minimal important difference in RQLQ). Sensitivity analysis with RQLQ and TSS6 scales confirmed the cut‐off value.

### 3.4 Comparison of outcomes in allergic rhinitis in children, adolescents and adults consulting specialists (2013)

In paper 4, in grass pollen‐allergic patients, a 4‐week multi‐centre, observational study was carried out in children (aged 6–11), adolescents and adults consulting specialist physicians.^9^, ^10^ In all age groups, the VAS score was strongly correlated with the weekly mean TSS score (Pearson's r: 0.79–0.88) and moderately correlated with the weekly mean RQLQ score (Pearson's r: 0.64–0.80). In moderate‐to‐severe grass pollen‐induced AR, symptom perception differed in children versus adolescents and adults. However, the assessments of treatment outcomes (using the TSS, VAS and RQLQ) were similar in all age groups. In an a priori sub‐analysis including WPAI‐AS, a multiple regression analysis indicated that the RQLQ score was a weak but statistically significant predictor of both impaired work/classroom productivity and daily activities.^10^

## 4 SELF‐ AND PHYSICIAN‐ADMINISTERED VAS ‘ASTHMA’ AND ‘RHINITIS’ ON PAPER AND PENCIL IN JAPAN (2009)

**FIGURE 2:**
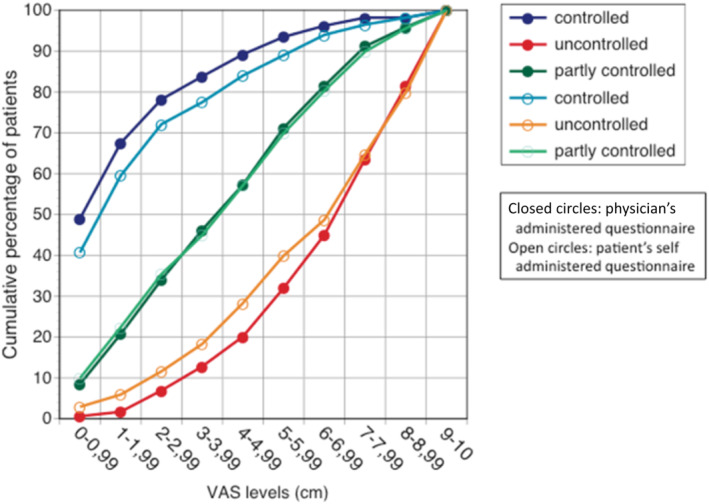
Distribution of GINA control categories relative to VAS levels.

In study 5, in Japan, 1910 physicians enrolled 29,518 patients with diagnosed and treated asthma.^18^ In total, 15,051 (51.0%) questionnaires were administered by physicians. Self‐ and physician‐administered questionnaires produced similar results. Median VAS asthma levels were of 4.75 (25%–75%: 1.85–7.00) in the self‐administered questionnaire versus 4.79 (1.90–7.18) in the physician‐administered questionnaire. Median VAS rhinitis levels were 5.49 (2.51–7.90) in the self‐administered questionnaire versus 5.60 (2.75–7.85) in the physician‐administered questionnaire. An a priori sub‐analysis found that the VAS was a strong predictor of GINA‐defined asthma.^19^ VAS asthma predicted levels of GINA‐defined control categories (the area under the ROC curve ranged from 0.704 to 0.837), particularly with the VAS cut‐offs of 1.50, 4.79 and 7.19. Similar results have been obtained using self‐ and physician‐administered questionnaires showing the consistency of the results (Figure 2).

**FIGURE 3:**
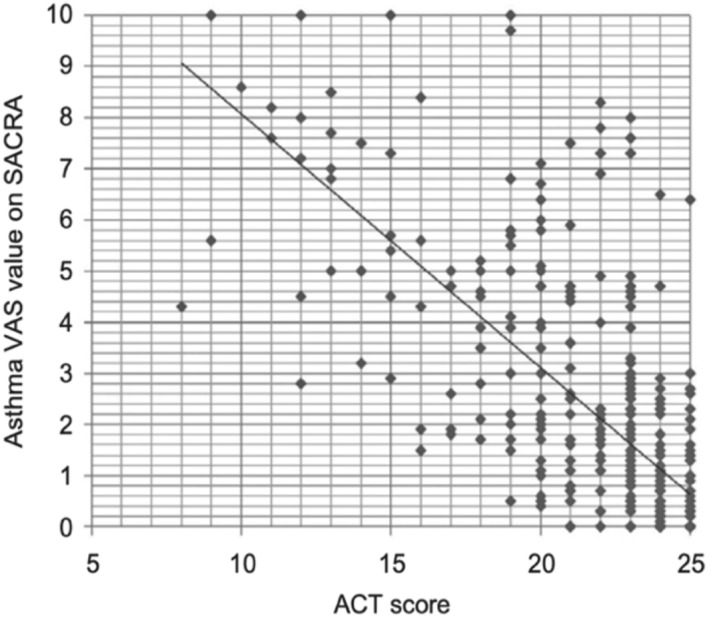
Correlation between ACT and VAS asthma (SACRA) (from^20^).

An a posteriori sub‐analysis was carried out in 420 asthmatic patients using self‐administered questionnaires,^20^ VAS asthma and the Asthma Control Test (ACT) score. It showed a strong correlation (rho = −0.70, *p* < 0.001) (Figure 3). For an ACT<19 (cut‐off for asthma control), most of the VAS asthma levels were over 20/100. However, for an ACT>19, many patients had high VAS asthma levels.

## 5 VAS ‘GLOBAL ALLERGY SYMPTOMS’ AND TOTAL SYMPTOM SCORE (TSS) USING AN ELECTRONIC DIARY IN 2 LARGE DB‐PC‐RCTS IN EUROPEAN AND CANADIAN SPECIALISTS (2008)

Two double‐blind, placebo‐controlled, randomised‐controlled trials (DB‐PC‐RCTs) were conducted to assess whether desloratadine was effective in intermittent or persistent AR against placebo.^21^, ^22^, ^34^, ^35^

**FIGURE 4:**
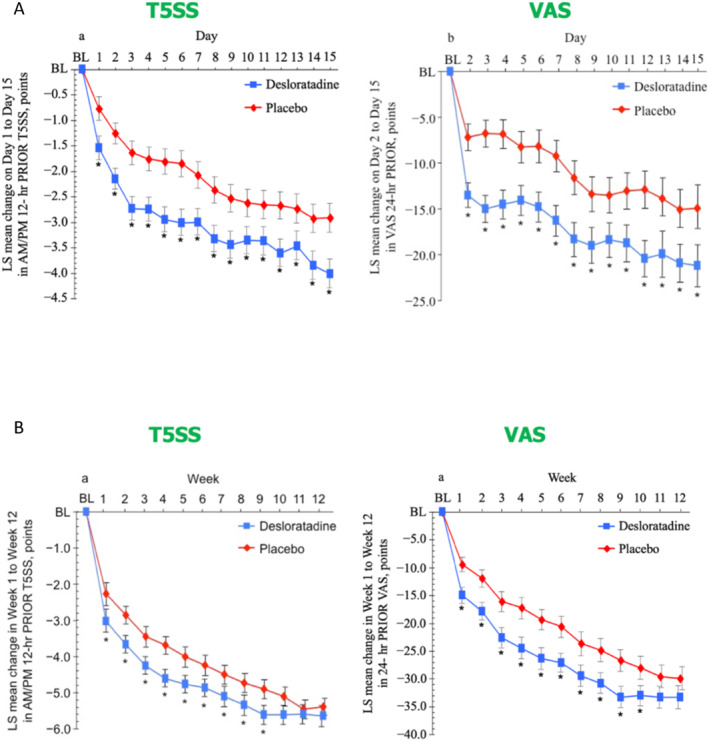
(A) Evolution of T5SS and VAS global allergy symptoms in patients with intermittent allergic rhinitis treated with desloratadine or placebo. (B) Evolution of T5SS and VAS global allergy symptoms in patients with persistent allergic rhinitis treated with desloratadine or placebo.

The first study assessed adolescents and adults with intermittent AR for 15 days^21^: desloratadine 5 mg once daily (*n* = 276) or placebo (*n* = 271). The primary endpoint, the AM/PM reflective five‐symptom VAS, was compared with T5SS and displayed similar change patterns (Figure 4A).

The second study assessed adolescents and adults with perennial AR under treatment with desloratadine 5 mg once daily (*n* = 360) or placebo (*n* = 356) over an 85‐day time period.^22^ The primary outcome was the AM/PM reflective TSS5 averaged over days 1–29. Similar change patterns were observed for the TSS5 and VAS (Figure 4B). The first 2 weeks of analyses were similar in intermittent and perennial AR for both TSS and VAS, showing the strength of both approaches.

## 6 VAS ‘RHINO‐CONJUNCTIVITIS SYMPTOMS’, TSS, TNSS AND TOSS IN AN ALLERGEN‐IMMUNOTHERAPY DP‐PC‐RCT IN EUROPE

A *post‐hoc* analysis^23^ was carried out from one pivotal DB‐PC‐RCT on immunotherapy with birch pollen.^36^ For each patient, TNSS, TOSS, TSS and VAS for rhinoconjunctivitis symptoms (VAS‐RS) were assessed daily and RQLQ was assessed weekly between 2 weeks before and 2 weeks after the anticipated start date of the pollen season. Data from 482 subjects, providing 2937 daily records, were analysed. Daily VAS‐RS was strongly correlated with the remaining daily PROMs and RQLQ score collected on the same day or in the respective weeks (Table 5). Similar results were obtained when considering the full pollen season or the 7 worst days of the pollen season.

**TABLE 5:** Correlations between daily reported patient‐reported outcome measures and RQLQ.

	Correlations with the RQLQ (95% CI)^a^	Correlations with the VAS‐RS (95% CI)^a^
VAS‐RS		
Single day	0.73 (0.69; 0.77)	‐
Weekly median	0.74 (0.70; 0.78)	‐
TNSS		
Single day	0.73 (0.69; 0.77)	0.76 (0.72; 0.80)
Weekly median	0.77 (0.73; 0.80)	0.77 (0.73; 0.80)
TOSS		
Single day	0.66 (0.61; 0.71)	0.66 (0.61; 0.71)
Weekly median	0.68 (0.63; 0.73)	0.67 (0.62; 0.72)
TSS		
Single day	0.77 (0.73; 0.80)	0.80 (0.73; 0.83)
Weekly median	0.81 (0.78; 0.84)	0.80 (0.73; 0.83)

*Note*: Confidence intervals around correlation are computed using Fisher's large sample formula.

Abbreviations: CI, Confidence interval; RQLQ, Rhinoconjunctivitis Quality of Life Questionnaire; TNSS, Total nasal symptom score; TOSS, Total ocular symptom score; TSS, Total symptom score; VAS‐RS, Visual analogue scale on rhino‐conjunctivitis symptoms.

^a^ Correlation coefficients adjusted for repeated measures.

## 7 MASK‐AIR^®^ STUDIES (2015‐)

**FIGURE 5:**
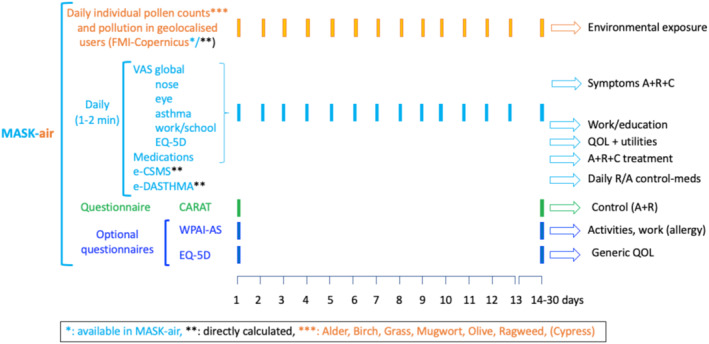
The MASK‐air^®^ app.

MASK (Mobile Airway Sentinel networK), the Phase 3 ARIA^33^, ^37^ initiative, is a flexible e‐platform for AR and asthma and includes the MASK‐air^®^ app, which is operational in 30 countries and 20 languages (Table S1). Over 63,000 users have been registered. A description of MASK‐air^®^ features is summarised in Figure 9. Its specifications have been published.^38^ (Figures 5 and S1).

MASK‐air^®^, a validated mHealth app (Medical Device regulation Class IIa), is a Good Practice of DG Santé on digitally enabled person‐centred care^39^ and a Best Practice of OECD (Organisation for Economic Co‐operation and Development) for Public Health guidelines in chronic diseases. The vision of MASK‐air^®^ has led to a strategic overview that was initiated by ARIA in 1999. It includes WHO‐associated projects,^33^, ^40^ EU grants and projects^41^, ^42^, ^43^, ^44^, ^45^, ^46^, ^47^ and four ARIA‐EAACI Task Forces.^26^, ^48^, ^49^, ^50^

Even though MASK‐air^®^ is not the only multilingual app in rhinology (a market‐research^51^ identified three such apps: AllergyMonitor, Pollen Austria and MASK‐air^®^), it is the best validated app for clinical practice and studies.

### 7.1 Methodological approach

MASK‐air^®^ has been validated using COSMIN guidelines and a series of methodologic studies.^52^ Limitations of MASK‐air have been clearly defined.^52^

The Technology Readiness Levels (TRLs)^53^ are usually eight or nine including PROMs for rhinitis and asthma, VAS, EQ‐5D, CARAT, e‐CSMS and e‐DASTHMA.

### 7.2 Patient's acceptability

Two qualitative studies were carried out by MADOPA (*Maintien en Autonomie à Domicile des Personnes Agées*, https://www.madopa.fr) in 2016 to better understand the patients' needs and expectations.^54^ Their comments were embedded in MASK‐air.

Five studies carried out in France (in preparation), Italy,^55^ Lithuania,^56^ Poland^57^ and Portugal (submitted) showed that patients have an overall positive appreciation of MASK‐air^®^ and proposed further improvements of the app.

### 7.3 Impact of age on MASK‐air^®^

Studies assessing MASK‐air^®^ data have traditionally included users ranging in age from 16 to over 90 years. Elderly users (≥65 years) can use the MASK‐air^®^ app after a short training period.^55^ We assessed 19,369 users <65 years (333,395 days) and 519 users ≥65 (15,650 days) from 24 countries.^58^ Days of users <65 and ≥65 years had overall similar clinical characteristics and asthma and rhinitis medication patterns. Comparing days from users 65–74 years of age with those ≥75, we observed small and moderate effect size measures in some variables, particularly in VAS eye and VAS asthma.

### 7.4 Daily MASK‐air^®^ PROMs

#### 7.4.1 Daily VASs

The selection and validation of MASK‐air^®^ VASs were based on studies using paper and pencil or e‐diaries: VASs assessing for global allergy, nasal, ocular and asthma symptoms (Table 2).^31^, ^59^ In addition, MASK‐air^®^ assessed the daily impact of the allergies or asthma on work^28^ or academic productivity^29^ by means of VASs. A generic VAS on QOL (EQ‐5D‐5L VAS) was also included.^25^, ^30^ Moreover, daily and personalised pollen counts and pollution data are available to complement VASs (Finish Meteorological Institute and Copernicus).^60^

**FIGURE 6:**
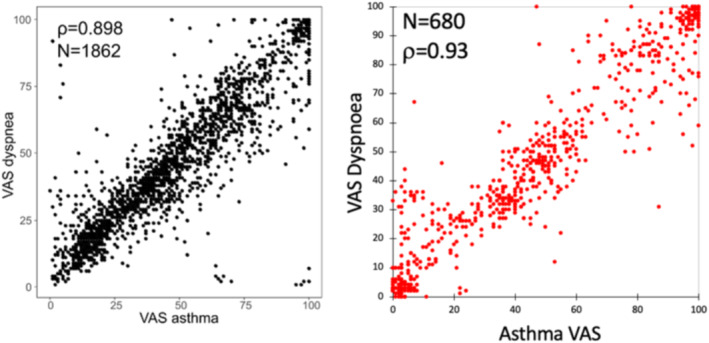
Correlation between VAS asthma and dysponea in two studies on severe asthma (from^31^, ^32^).

In MASK‐air^®^, VAS asthma is highly correlated with VAS “dyspnoea” that was subsequently removed (Figure 6).^31^, ^32^

Small airways represent an important feature of asthma, and VAS asthma has been correlated with impulse oscillometry (IOS) in symptomatic patients.^61^

A pilot trial evaluated the usefulness of the MASK‐air^®^ app in improving rhinitis control in 262 patients with AR and asthma.^62^ There was a strong significant correlation between VAS asthma and ACT (Pearson coefficient: −0.79). In agreement, VAS asthma and ACT have shown correlation in a recent Turkish study (unpublished data).

#### 7.4.2 VAS cut‐off values

In MASK‐air, cut‐offs were calculated using two different approaches in 395,223 days from 23,201 users: one based on the VAS percentiles and another based on VAS work and EQ‐5D levels (Table 6).^63^

**TABLE 6:** MASK‐air cut‐off levels (/100) (from^63^).

	Arbitrary^5^	Outcome‐oriented^63^	
		VAS global, nose, asthma	VAS eye
Full control	0	0	0
Control	1–19	1–19	1–12
Partial control	20–49	20–35	13–35
No control	≥50	≥35	≥35

#### 7.4.3 Concurrent validity of VAS in MASK‐air^®^

VAS asthma was studied using MASK‐air^®^ data. Correlations between VAS asthma and other MASK‐air^®^ daily reported PROMs were studied in severe asthmatic patients with nasal symptoms. Strong correlations were found between VAS asthma and other measures (Table 7).^31^, ^32^

**TABLE 7:** Correlation coefficients between different PROMs in severe asthma (from^31^).

	*N* observations	Spearman correlation coefficient (95% CI)	Repeated measures correlation coefficient (95% CI)^64^
VAS asthma versus VAS dyspnoea	1862	0.898 (0.879; 0.915)	0.713 (0.690; 0.735)
VAS asthma versus VAS global	4822	0.767 (0.750; 0.784)	0.544 (0.524; 0.564)
VAS asthma versus VAS nose	4822	0.755 (0.738; 0.771)	0.465 (0.443; 0.487)
VAS asthma versus VAS work	1840	0.768 (0.739; 0.793)	1.658 (0.631; 0.683)

#### 7.4.4 Combined electronic daily symptom‐medication scores based on VAS

Validated daily electronic combined symptom‐medication scores (CSMSs) are needed to investigate the effects of AR or asthma treatments. MASK‐air^®^ includes a medication list with all medications customised by countries. Combining medications and VAS allows the computation of electronic daily scores for allergic diseases (allergy e‐CSMS)^26^ and asthma (e‐DASTHMA).^48^

##### Allergy e‐CSMS

**FIGURE 7:**
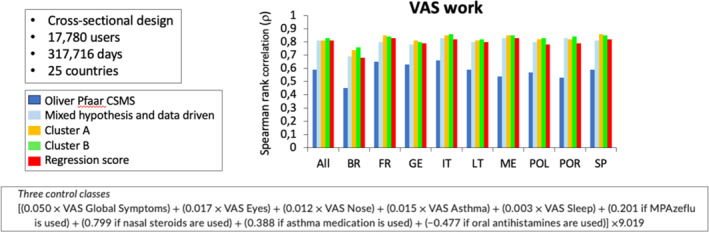
Allergy e‐CSMS compared to VAS work (from^26^). BR, Brazil; FR, France; GE, Germany; IT, Italy; LT, Lithuania; ME, Mexico; POL,Poland; POR, Portugal; SP, Spain.

In 317,176 days of MASK‐air^®^ use from 17,780 users aged 16–90 years in 25 countries, allergy e‐CSMS was computed following cluster analysis, regression models or factor analysis (Figure 7). The selected CSMS displayed high accuracy (capacity of discriminating different levels of rhinitis control), and medium‐high validity and reliability, rendering it as a candidate for primary endpoints in future rhinitis trials. Results were similar in different countries, showing the transferability and cultural adaptation of the CSMS. e‐CSMS has been used in several MASK‐air^®^ studies.^26^, ^58^, ^65^, ^66^, ^67^, ^68^, ^69^

##### e‐DASTHMA

**FIGURE 8:**
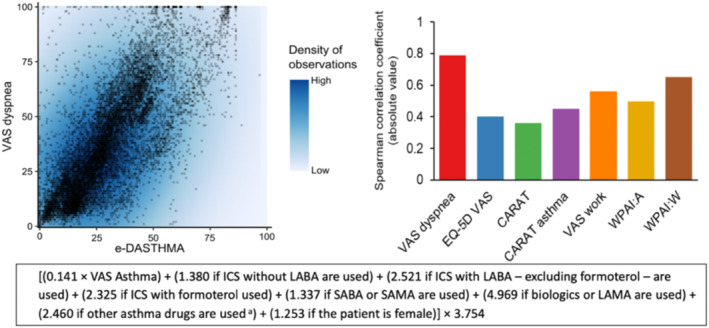
Results between the best performing e‐DASTHMA and comparators (from^48^).

An electronic daily asthma control score, e‐DASTHMA,^48^ has been developed in a cross‐sectional study (35,635 days of MASK‐air^®^ data, 1662 users). The developed score was strongly correlated with VAS dyspnoea and moderately correlated with other outcomes. It displayed high test‐retest reliability and moderate‐to‐high responsiveness (Figure 8).

An external validation of e‐DASTHMA was performed using an independent cohort of patients with physician‐diagnosed asthma (INSPIRERS).^70^ e‐DASTHMA was strongly correlated with the GINA classification of control. e‐DASTHMA has been used in MASK‐air^®^ studies.^71^

### 7.5 Other PROMs

Additional questionnaires are included in MASK‐air^®^: Control of Allergic Rhinitis and Asthma Test (CARAT, a PROM that assesses the level of control of both asthma and AR using a single tool over a period of 4 weeks),^72^ WPAI‐AS^73^, ^74^ and EQ‐5D (Figure 7). However, other control tests can also be included in MASK‐air^®^.

### 7.6 Impact of VAS levels

#### 7.6.1 Work productivity

**FIGURE 9:**
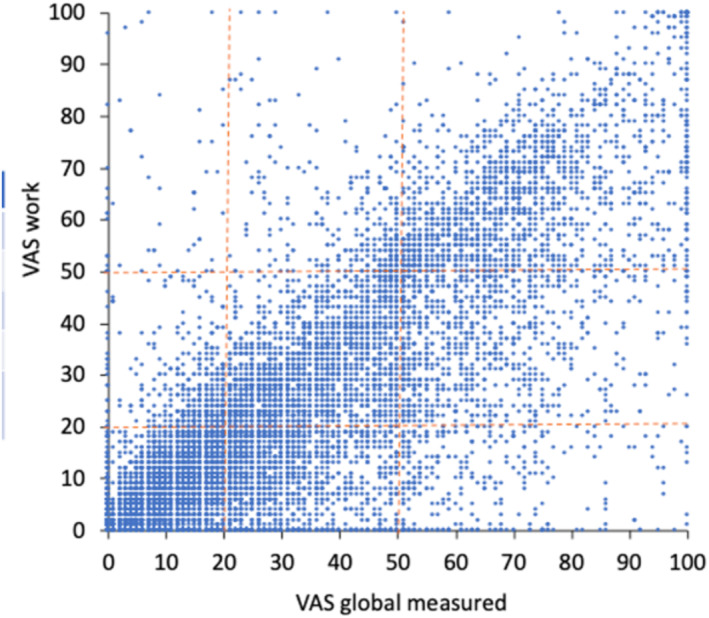
Correlation between VAS work and global allergy symptoms (from^77^ *N* = 16,925 days).

Rhinitis has a significant impact on work productivity.^75^ VAS work and WPAI‐AS have been used in a series of concurrent validity studies (Table 8 and Figure 9). A significant correlation has been found between VAS work and WPAI‐AS.

**TABLE 8:** Correlations using Spearman's test between VAS work and other VASs.

	*N* days (study)	VAS global	VAS nose	VAS asthma	VAS eye
Bousquet^76^	6120 (CS)	0.83	0.80	0.57^a^	0.70
Bousquet^77^	16,925 (CS)	0.82	0.77	0.60^a^	0.69
Bedard^28^	98,303 (CS)	0.73	0.68	0.45^a^	0.56
Sousa‐Pinto^78^	149,732 (CS)	0.81	0.78		
Sousa‐Pinto^31^	1840 (L)			0.79^b^	
Benfante^22^	1222 (L)			0.85^b^	

Abbreviations: CS, cross‐sectional; L, longitudinal.

^a^ The entire population of asthmatic and non‐asthmatic patients was compared.

^b^ In severe asthmatic patients only.

#### 7.6.2 Education

Allergic diseases have a significant impact on academic performance and a MASK‐air^®^ study compared VAS on allergy symptoms with VAS academic productivity.^29^ In 13,454 days, there was a significant correlation between VAS education and VAS global (rho = 0.70) and VAS nose (rho = 0.66). The WPAI‐AS provided data on the impact of allergies on school performance in 125 weeks with 6–7 days of reporting. There was a significant correlation between WPAI‐AS and VAS education (rho = 0.80). Similar results were obtained when correlations were assessed using repeated measures correlation coefficients.

#### 7.6.3 EQ‐5D‐5L

**FIGURE 10:**
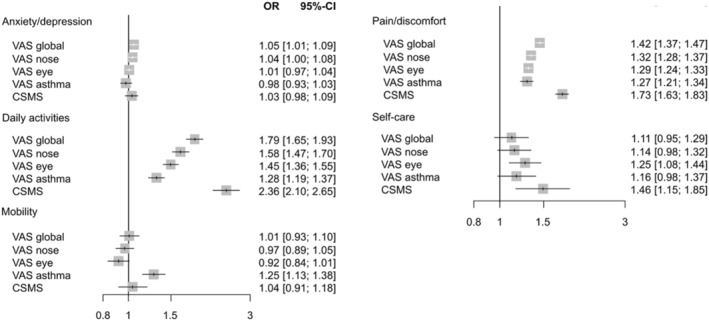
Association of VAS or CSMS with EQ‐5D‐5L domains in a multivariate analysis (from^66^).

Very few studies on allergic rhinitis and asthma have assessed each of the five EQ‐5D domains individually. A cross‐sectional MASK‐air^®^ study assessed the association between rhinitis or asthma control and the different EQ‐5D‐5L domains in 5354 days from 3092 different users.^66^ Worse control of rhinitis, conjunctivitis or asthma (median VASs and CSMS) was associated with an impairment of EQ‐5D levels. Mobility was particularly associated with VAS asthma (Figure 10). The expected differences in the EQ‐5D ‘mobility’ domain between VAS asthma and the other comparators^79^ strengthen the validity of VAS asthma. On the other hand, VAS asthma is less well correlated with the EQ‐5D ‘daily activities’ domain, in accordance with the results of the European Community Respiratory Health Study.^79^

### 7.7 Intra‐rater reliability

Between 2412 (“VAS work”) and 5827 (“VAS nose” and “VAS eyes”) days with more than one daily monitoring VAS input provided by the same user were recorded.^78^ For all VASs, more than 50% of the days had no differences in the first and second values provided within the same day. Differences between the first and second daily values differing by more than 10% ranged between 11.2% (“VAS asthma”) and 24.4% (“VAS nose”). The intraclass correlation coefficients (ICC) were over 0.87 (“VAS global allergy symptoms”). Similar results were observed when analysing data from MASK‐air^®^ European users only or by taking into account the first and last daily measurements. Overall, these results indicate that the MASK‐air^®^ VASs display a high intra‐rater reliability.

### 7.8 Test‐retest reliability

Using clinical stability defined according to several outcomes, the test‐retest reliability of daily monitoring VASs was assessed based on data from 102 (“VAS work”) to 270 (“VAS global allergy symptoms,” “VAS nose,” “VAS eyes” and “VAS asthma”) patients (Table 9).^78^ Overall, results indicate that MASK‐air^®^ VASs display a high test‐retest reliability, with ICC being always above 0.74. That is, VAS levels tend to display no or small changes in clinically stable patients.

**TABLE 9:** Test‐retest analyses from 25 countries (from^78^).

VAS	EQ‐5D VAS		CARAT		Work VAS	
	*N* users	ICC (95% CI)	*N* users	ICC (95% CI)	*N* users	ICC (95% CI)
Global allergy symptoms	270	0.75 (0.62–0.82)	134	0.745 (0.64–0.82)	5761	0.85 (0.83–0.86)
nose	270	0.77 (0.71–9.81)	134	0.77 (0.67–0.84)	5763	0.84 (0.83–0.84)
eye	270	0.75 (0.68–0.80)	134	0.74 (0.63–0.82)	5763	0.83 (0.82–0.84)
asthma	270	0.86 (0.82–0.89)	134	0.84 (0.775–0.87)	5763	0.88 (0.87–0.88)
work	102	0.82 (0.74–0.88)	35	0.82 (0.605–0.915)	‐	

### 7.9 Ability to change

A pilot trial evaluated the usefulness of the MASK‐air^®^ app in improving rhinitis control in 262 patients with AR.^62^ VAS global changed similarly to TNSS.

In a quasi‐experimental study in Greece, CARAT and MASK‐air^®^ provided complementary information on AR symptom control, possibly mirroring differences in time periods assessed by these two tools (4 weeks vs. daily assessment).^80^

Using clinical change, defined according to several outcomes, the responsiveness of the daily monitoring of VASs was assessed based on data from 27 to 108 users. Meaningful low‐moderate effect size measures were observed for all analyses, indicating that MASK‐air^®^ VASs may have moderate responsiveness (i.e., meaningful change in clinically unstable patients).

### 7.10 Transferability and cultural acceptability

MASK‐air^®^ data are derived from up to 27 countries. When results were provided for each of the countries, there was a high correlation between them, including Brazil and Mexico. It can be concluded that MASK‐air^®^ can be used in many countries and that the results are similar. However, for every new study, transferability would need to be confirmed since some questions may not be fully relevant in all countries.

## 8 CONCLUSIONS

### 8.1 Pre‐MASK‐air^®^ studies

For construct validity, in patients with rhinitis and/or asthma, the different VASs used have been shown to be highly correlated to RQLQ and total symptom scores for rhinitis and asthma control scores or to the GINA classification of control. Cut‐off VAS values have been identified. The ability to detect a change was also found and correlated with TNSS or TSS and RQLQ.

### 8.2 MASK‐air^®^ studies

Similar findings have been observed with MASK‐air^®^ for daily PROMs. More data are needed for the ability to change. Importantly, similar results were found in different countries, indicating the transferability and cultural acceptability of MASK‐air^®^ PROMs across countries.

### 8.3 Clinical relevance of MASK‐air^®^ PROMs and future studies

Simple PROMs that can be easily translated into several languages and do not need cultural adaptation are available in an electronic form in MASK‐air^®^. They are based on VASs that were found to be of clinical relevance. The daily electronic combined symptom‐medication scores represent an improvement over VAS as they represent both PROMs and medications. However, these tools need to be tested to determine their relevance in the stratification of severe patients and the cost‐effective management of rhinitis and/or asthma to provide recommendations for policy makers. Preliminary work has been developed to assess how MASK‐air^®^ PROMs can be used for patient stratification. In fact, we assessed complete months in which patients under inhaled corticosteroids had reported VAS asthma and e‐DASTHMA levels, identifying groups of patients who would most probably benefit from treatment scaling up versus those who would benefit from increased adherence (Sousa‐Pinto et al., in preparation). This can support efforts to increase the efficiency in the management of allergic diseases. Finally, these PROMs are currently being tested for their value in the diagnosis of under‐recognised patients.

**Box 1 Differences between total symptom scores in rhinitis**

1

**TNSS**: Total nasal symptom score, currently typically measured with 4 symptoms (rhinorrhoea, nasal congestion, sneezing and nasal itching), with each symptom being rated on a 0–3 scale. Variations with 3 or 5 symptoms exist and have been classically used.
**TOSS**: Total ocular symptom score, currently typically measured with 3 symptoms (eye itching/burning, tearing/watering and redness), with each symptom being rated on a 0–3 scale. Variations in the number of symptoms exist (typically up to 3)
**TSS**: Total symptom score combining TNSS and ocular symptoms or TOSS

## AUTHOR CONTRIBUTIONS

**Jean Bousquet**: Conceptualisation; writing—original draft; writing—review and editing; supervision; methodology; validation; resources; visualisation; formal analysis; data curation. **Bernardo Sousa‐Pinto**: Writing—review and editing; supervision. **Josep M. Anto**: Supervision; writing—review and editing. **Anna Bedbrook**: Supervision; writing—review and editing. **Wienczyslawa Czarlewski**: Writing—review and editing; supervision. **Ignacio J. Ansotegui**: Writing—review and editing. **Karl‐C. Bergmann**: Writing—review and editing. **Fulvio Braido**: Writing—review and editing. **Luisa Brussino**: Writing—review and editing. **Lorenzo Cecchi**: Writing—review and editing. **Claudia Chaves Loureiro**: Writing—review and editing. **Alvaro A. Cruz**: Writing—review and editing. **Philippe Devillier**: Writing—review and editing. **Alessandro Fiocchi**: Writing—review and editing. **Bilun Gemicioglu**: Writing—review and editing. **Tari Haahtela**: Writing—review and editing. **Juan Carlos Ivancevich**: Writing—review and editing. **Ludger Klimek**: Writing—review and editing. **Marek Kulus**: Writing—review and editing. **Piotr Kuna**: Writing—review and editing. **Maciej Kupczyk**: Writing—review and editing. **Violeta Kvedariene**: Writing—review and editing. **Desiree E. Larenas‐Linnemann**: Writing—review and editing. **Gilles Louis**: Writing—review and editing. **Renaud Louis**: Writing—review and editing. **Michael Makris**: Writing—review and editing. **Mario Morais‐Almeida**: Writing—review and editing. **Marek Niedoszytko**: Writing—review and editing. **Ken Ohta**: Writing—review and editing. **Markus Ollert**: Writing—review and editing. **Nikolaos Papadopoulos**: Writing—review and editing. **Vincenzo Patella**: Writing—review and editing. **Benoit Pétré**: Writing—review and editing. **Oliver Pfaar**: Writing—review and editing. **Francesca Puggioni**: Writing—review and editing. **Santiago Quirce**: Writing—review and editing. **Frederico S. Regateiro**: Writing—review and editing. **Nicolas Roche**: Writing—review and editing. **Philip W. Rouadi**: Writing—review and editing. **Boleslaw Samolinski**: Writing—review and editing. **Joaquin Sastre**: Writing—review and editing. **Florence Schleich**: Writing—review and editing. **Nicola Scichilone**: Writing—review and editing. **Luis Taborda‐Barata**: Writing—review and editing. **Sanna Toppila‐Salmi**: Writing—review and editing. **Arunas Valiulis**: Writing—review and editing. **Ilgim Vardaloglu Koyuncu**: Writing—review and editing. **Maria Teresa Ventura**: Writing—review and editing. **Arzu Yorgancioglu**: Writing—review and editing. **Joao A. Fonseca**: Supervision; writing—review and editing. **Torsten Zuberbier**: Supervision; writing—review and editing.

## CONFLICT OF INTEREST STATEMENT

The authors declare no conflicts of interest.

## HUMAN STUDIES AND SUBJECTS

All reviewed studies conformed to the Declaration of Helsinki.

## CLINICAL TRIAL REGISTRATION

Some reported studies were registered, and the registration numbers are in the published papers.

## Supporting information

Supporting Information S1

## Data Availability

Data sharing not applicable to this article as no datasets were generated or analysed during the current study.
