# Supplementary material for: Concurrent validity, cut‐offs and ability to change of patient‐reported outcome measures for rhinitis and asthma in MASK‐air®
Source: Clin Transl Allergy. 2024 Sep 23;14(9):e12390. doi: 10.1002/clt2.12390 (PMC11419846; doi:10.1002/clt2.12390)
Supplement: Supplementary file 1 — Supporting Information S1 [file CLT2-14-e12390-s001.docx]

**ANNEX 1: Development of MASK-air®**

1. **Customer needs:** Using visual analogue scales (VASs), users can track their symptoms for AR (global allergy impact, nose, eyes), asthma (VAS asthma) as well as work, school productivity^101^ and EQ-5D.^25^ Then users record their medications for R, conjunctivitis and asthma using a regularly updated treatment scroll list including all medications customised for each country.

MASK-air^®^ is combined with prediction on allergen season and air quality (POLLAR: Impact of air POLLution on Asthma and Rhinitis, EIT Health-funded project) using COPERNICUS data. ^56^

The MASK-air^®^ app has been designed to avoid any missing data in the secure database.

1. **Intended use:** To help patients with AR and/or asthma monitor their symptoms and to inform them on their control. The app does not suggest any specific treatment decisions and is a supportive tool.
2. **Intended users:** Patients affected by AR. The app is not intended for minors (children under 16 or under the age of digital consent depending on the country). However, after ethical agreement and parental approval, the app can be used by minors. KyoPro has also been developed for children.
3. **Software (SW) operation, functionality and technical description:** MASK-air^®^ is a mobile phone app freely available for Android and iOS. The MASK-air website (https://www.mask-air.org/data) shares data collected on the users’ phone with his/her healthcare provider during the appointment. The app consists of (i) VASs and a medication scroll list, (ii) a QR code reader module, (iii) my profile, (iv) reminders and (v) additional questionnaires: EQ-5D, ^25^ WPAI-AS (Work Productivity and Activity^65^) and CARAT (Control of Rhinitis and Asthma Test). ^104^
4. **Labelling information app.** The software (SW) MASK-air^®^ was initially marketed as the “Allergy App”. ^105^ The SW application itself identifies the legal manufacturer (Peercode, https://www.peercode.nl/nl/welkom/), provides screens with contact details and carries the relevant CE mark. It includes an integrated Terms of Use (ToU)/Privacy Policy section that covers all applicable regulatory required labelling content, such as specific operating instructions prior to use and any potential use related advice. ^80^ No specific warnings, separate non-digital labelling documentation or Instructions for Use are provided to the user.

The ToU information contains the instructions that are relevant prior and during use and that will be available at all times in the app store(s) and in the app and exchange-website itself. ^80^

After downloading the app and prior to use, the user has to accept the ToU containing the privacy policy. With acceptance of the ToU, the information is considered understood and accepted.

1. **MASK-air® is available in country relevant languages:** The EU CE marking is a Class IIa compliant product under the EU MDR, but will be put on the market at a later stage. The labelling will remain EU MDD compliant until such a time. Country specific deviations to the core labelling will be addressed in the Labelling documentation. It is operational in 28 countries and 19 languages (Figure 3 online).
2. **Labelling information on the physician’s exchange website:** On the bottom banner, a link to the Privacy Policy and ToU is shown. The CE mark with a notified body number is shown when applicable. Clicking on the CE symbol opens the additional information such as legal manufacturer and contact details.
3. **Labelling information peercode.nl website** A link is provided to the ToU/ Privacy policy and to the Labelling on the mask-air.org website. Over 58,000 users have been registered.
4. **Secure database**. The secure database is shown in Figure S1.

**Figure S1: Secure database**

**ANNEX 2: Major achievements of MASK-air^®^**

**Table S1: Major achievements of MASK-air** (updated from^47^)

|  | **Study name** | **Ref** | **Study type** | **N users** | **N days** | **N countries** |
| --- | --- | --- | --- | --- | --- | --- |
| **Baseline characteristics** | | | | | | |
| 25 | Pilot study of mobile phone technology in AR in European countries. The MASK-rhinitis study | ^105^ | Obs, CS | 3,260 | NA | 20 |
| 26 | Treatment of AR during and outside the pollen season using mobile technology. A MASK study | ^106^ | Obs, CS | 9,035 | 70,286 | Europe  18 |
| **Symptoms in the diagnosis and control of asthma** | | | | | | |
| 27 | Impact of asthma symptoms on QOL** | ^107^ | Obs, CS | 171 | 171 | Belgium |
| 28 | Impact of CARAT questions on the diagnosis and control of asthma | Submitted | Obs, CS | 951 | 2,153 | 25 |
| **Phenotype of allergic diseases and asthma** | | | | | | |
| 29 | Daily allergic multimorbidities | ^108^ | Obs, CS | 4,210 | 32,585 | 19 |
| 30 | Clusters of asthma and rhinitis | ^109^ | Obs, CS | 8,075 | 297,169 | 26 |
| 31 | Trajectories of allergic rhinitis | ^110^ | Obs, L | 2,590 | 113,239 16,177 wks | 26 |
| 32 | Rhinitis associated with asthma is distinct from Rhinitis alone | Submitted | Obs, CS+L | 3,797 | 256,839 | 27 |
| **Adherence to treatment of AR using mobile technology** | | | | | | |
| 33 | Adherence to treatment of AR using mobile technology | ^111^ | Obs, CS | 6,949 | NA | 21 |
| **Real-world data in allergic rhinitis** | | | | | | |
| 34 | Treatment of AR using mobile technology with real-world data: The MASK observational pilot study | ^112^ | Obs, CS | 2,871 | 39,634 | 25 |
| 35 | Mobile technology offers novel insights on control and treatment of AR. The MASK study | ^113^ | Obs, CS | 9,122 | 112,054 | 23 |
| 36 | Treatment of AR during and outside the pollen season using mobile technology. A MASK study | ^106^ | Obs, CS | 9,035 | 70,286 | Europe  18 |
| 37 | Comparison of rhinitis treatments using MASK-air^®^ data considering the Minimal Important Difference | ^114^ | Obs, CS | 10,860 | 269,837 | 28 |
| 38 | Differences in behavioural patterns in AR medication in Europe: A study using MASK-air^®^ real-world data | ^115^ | Obs, CS | 13,122 | 222,024 | Europe  18 |
| **Real-world data in asthma** | | | | | | |
| 39 | Longitudinal severe asthma pilot study | ^116^ | Obs, CS | 13 | 1,250 | Italy |
| 40 | Treatment of asthma using mHealth real-world data: The MASK-air observational study | Submitted | Obs, CS | 3,229 | 70,270 | 27 |
| 41 | Adherence to ICS/LABAs in asthma | Submitted | Obs, L | 866 | 26,396  6,444 wks | 27 |
| **Impact of allergic diseases** | | | | | | |
| 42 | Academic productivity in AR: A MASK-air^®^ direct data cross-sectional study | ^117^ | Obs, CS | 1,970 | 13,454 | 28 |
|  | | | | | | |
| **Clinical trials** | | | | | | |
| 43 | Validation of the MASK-air App for assessment of AR | ^87^ | RCT | 267 | 7,500 | Spain |
| 44 | Effect of nasal irrigation on AR control in children; complementarity between CARAT and MASK outcomes | ^103^ | RCT | 76 | NA | Greece |
| **Allergen immunotherapy** | | | | | | |
| 45 | Effect of AIT in the MASK-air® study: proof-of-concept analysis | ^118^ | Obs, CS | 17,780 | 317,176 | 25 |
| 46 | Allergen AIT in MASK-air users in real-life: results of a Bayesian mixed-effects model | ^119^ | Obs, CS | 1,093 | 42,756 | 25 |
| 47 | Daily improvement of allergy control by sublingual AIT: A MASK-air^®^ cross-sectional study | Submitted | Obs, CS | 217 | 4,726 | 14 |
| **Aerobiology and air pollution** | | | | | | |
| 48 | POLLAR | ^120^ | Obs, CS | 3,323 | 36,440 | 15 |
| *49* | *POLLAR** | *^121^* | *Review of methods used in POLLAR and embedded in MASK-air* | | | |

Obs: Observational, RCT: randomised controlled trial, CS: cross-sectional, L: longitudinal, NA: not available

AIT: Allergen immunotherapy, AR: Allergic Rhinitis, CARAT: Control of Rhinitis and Asthma Test, LABA: Long-acting ß2 agonist, ICS: Inhaled corticosteroids, POLLAR: Impact of air POLLution on Asthma and Rhinitis.

*: Review carried out for MASK-air, **: Paper related to MASK-air
